# DIFFERENT PROTOCOLS OF POSTCONDITIONING DOES NOT ATTENUATE MESENTERIC ISCHEMIA-REPERFUSION INJURY AFTER SHORT-TERM REPERFUSION

**DOI:** 10.1590/0102-6720201700010008

**Published:** 2017

**Authors:** Marcus Vinicius Henriques BRITO, Edson Yuzur YASOJIMA, Andressa Abnader MACHADO, Matheus Paiva Pacheco Reis SILVEIRA, Renan Kleber Costa TEIXEIRA, Vitor Nagai YAMAKI, Felipe Lobato da Silva COSTA

**Affiliations:** 1 Laboratory of Experimental Surgery, Faculty of Medicine, State University of Pará - UEPA, Belém, PA, Brazil.

**Keywords:** Mesenteric vascular disease, Small intestine, Post-conditioning, Rats.

## Abstract

**Background:**

Mesenteric ischemia is a challenging diagnosis. Delay in diagnosis can lead to extent bowel necrosis and poor outcomes. Ischemia and reperfusion syndrome plays an important role in this scenario.

**Aim:**

To access effects of different post-conditioning cycles on mesenteric ischemia-reperfusion syndrome.

**Method:**

Twenty-five rats were assigned into five groups: Sham, used to establish normal parameters; control group, submitted to mesenteric ischemia for 30 min; in groups GP3, GP1 and GP30, ischemia was followed by post-conditioning protocol, which consisted of 1 cycle of 3 min (GP3), 3 cycles of 1 min (GP1) or 6 cycles of 30 s (GP30), respectively. Ileum samples were harvested after one hour of reperfusion. Intestinal mucosal injury was evaluated through histopathological analysis.

**Results:**

The average of mesenteric injury degree was 0 in the sham group, 3.6 in the control group, 3.4 in GP3, 3.2 in GP1, and 3.0 in GP30; villous length average was 161.59 in sham group, 136.27 in control group, 135.89 in GP3, 129.46 in GP1, and 135.18 in GP30. Was found significant difference between sham and other groups (p<0.05); however, there was no difference among post-conditioning groups.

**Conclusion:**

Post-conditioning adopted protocols were not able to protect intestinal mucosa integrity after mesenteric ischemia and short term reperfusion.

## RESUMO

**Racional:**

O desfecho satisfatório na abordagem cirúrgica da obesidade deve contemplar, além da perda de peso, alteração significativa nas comorbidades preexistentes e na qualidade de vida dos pacientes.

**Objetivo:**

Avaliar a qualidade de vida no pós-operatório tardio de pacientes submetidos à cirurgia de gastrectomia vertical por videolaparoscopia.

**Métodos:**

Foi aplicado o questionário "Bariatric Analysis and Reporting Outcome System" (BAROS) em pacientes submetidos à gastrectomia vertical por videolaparoscopia.

**Resultados:**

Foram avaliados 47 pacientes, entre 21 e 60 anos de idade. O IMC médio antes da operação era 43,06±5,87 kg/m². A média percentual de redução do excesso de peso após foi de 85,46±23,6%. A pontuação obtida pelos pacientes no questionário sobre a melhora na qualidade de vida evidenciou resultado excelente (36,17%), ótimo (40,43%), bom (21,28%) e razoável (2,13%). Houve melhora clínica após a operação em todas as comorbidades investigadas.

**Conclusão:**

A perda de peso foi fundamental para a melhoria na qualidade de vida e proporcionou resolução ou a melhora clínica em todas as comorbidades investigadas.

## INTRODUCTION

Mesenteric ischemia is a challenging diagnosis. Delay in doing it can lead to extent bowel necrosis and poor outcomes; carrying various consequences after surgery such as diarrhea, poor absorption, short bowel syndrome and death. Ischemia and reperfusion syndrome plays an important role in this scenario^9^. 

During ischemia there is Na^+^/K^+^pump failure, Ca^2+^ influx, acidic substances formation, promoting endothelial injury, increasing permeability of the microcirculation and tissue edema that activates inflammatory response^13^. However, tissue damage is not limited to ischemia. When tissue oxygenation is restored, tissue damage is worsened by the reperfusion injury that is even more deleterious than ischemia^2^ ^,^ ^10^ ^,^ ^11^ ^,^ ^24^. 

Aiming to minimize deleterious effects of ischemia and reperfusion injury, Murry et al.^14^ introduced the concept of ischemic preconditioning, which consists of short periods of ischemia followed by short reperfusion, before a longer ischemic phase, in order to induce organ tolerance to the ischemia-reperfusion injury^14^ ^,^ ^19^. Several clinical and experimental studies proved the protective effect of the ischemic preconditioning, as well as, its ability to reduce tissue damage^7^ ^,^ ^15^ ^,^ ^17^. However, in certain clinical settings, it is often difficult to foresee when ischemia will occur, limiting the preconditioning applicability^19^.

In 2003 Zhao et al.^25^ started the concept of ischemic post-conditioning, which consists in performing short cycles of ischemia followed by short cycles of reperfusion, immediately after the ischemia, and before the permanent reperfusion. Experimental studies have shown that ischemic post-conditioning is effective on preventing ischemia-reperfusion injury in several tissues, having a similar effect to ischemic pre-conditioning^5^ ^,^ ^12^ ^,^ ^20^. However, when analyzed in a hyper acute phase, post-conditioning was not able to reduce the damage caused by ischemia and reperfusion syndrome^1^. 

Thus this study intends to investigate the effects of different post-conditioning cycles on induced-mesenteric ischemia-reperfusion injury in a short-term reperfusion period.

## METHODS

**FIGURE 1:**
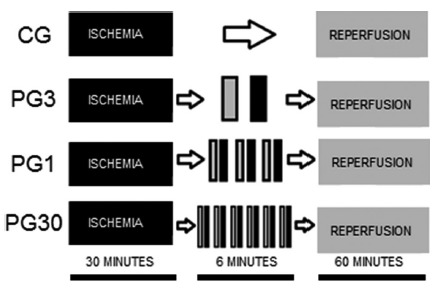
Diagram illustrating the post-conditioning protocols

This study was approved by the Ethics Committee for the Use of Animals of the State University of Pará - UEPA, protocol 22/10. Twenty five male Wistar rats (*Rattus norvegicus*) obtained from the Animal Colony of the Experimental Surgery Laboratory of UEPA were used. They weighed 200-240 g and were kept in a controlled environment with food and water ad libitum. The animals were randomly assigned into five study groups (n=5), according to the protocol (Figure 1): 1) sham group (SHAM), same surgical procedure as in remaining groups was performed, but no intestinal ischemia was induced; 2) control group (CG), intestinal ischemia was induced for 30 min followed by reperfusion without any form of conditioning; 3) post-conditioning group 3 min (GP3), intestinal ischemia was treated by 1 cycle of reperfusion interspersed by 1 cycle of ischemia, lasting 3 min each; 4) post-conditioning group 1 min (GP1), intestinal ischemia was treated by 3 cycles of reperfusion interspersed by 3 cycles of ischemia, lasting 1 min each; 5) post-conditioning group 30 s (GP30), intestinal ischemia was treated by 6 cycles of reperfusion interspersed by 6 cycles of ischemia, lasting 30 s each.

All surgical procedures were performed under anesthesia (ketamine-70 mg/kg and xylazine -10 mg/kg, intraperitoneally). Midline laparotomy was performed. During all procedure, the bowel was protected by gauze with heated saline. The mesenteric artery was identified and dissected, and it was occluded by a vascular clamp for 30 min. After the artery was clamped, the small intestine was repositioned inside the abdominal cavity and the skin was closed with 5-0 nylon stitches.

After the ischemic cycle, the abdominal cavity was reopened through removal of the stitches. Vascular clamp was taken off, starting the reperfusion period, lasting 60 min. In GP3, GP1 and GP30 groups, the ischemic post-conditioning were performed according the prior protocol described. After postconditioning protocol, abdomen was again closed with continuous 5-0 nylon stitches until the end of reperfusion period.

After reperfusion, the animals were euthanized by intravenous KCl injection. Immediately after, a 3 cm ileum sample distant 5 cm from the cecum valve was harvested. This fragment was fixed in 10% buffered formaldehyde, and underwent histological processing; the slides were stained with H&E and examined under light microscopy to determine the degree of injury based on the scale of Chiu et al.^4^ and villous length measurements were done by measuring ten well-oriented villous (cut across its length) in each of the animals.

### Statistical analysis

ANOVA test was used to compare villous length; Kruskal-Wallis test, to compare the histopathological results, and the Pearson's Linear Correlation test to determine whether there was a relationship between the score of intestinal injury and villous length. Significance level of 5% to reject the null hypothesis was adopted.

## RESULTS

During the procedure no animal died or resuscitation maneuvers were performed. The mean score of intestinal injury is demonstrated on Table 1. Sham group showed a normal mucosa, and there was significant difference between this group to all others who underwent ischemia; among the other groups there was no significant difference (p>0.05).

**TABLE 1:** Mean and standard deviation of intestinal injury score according the groups

Group	SG*	CG	PG3	PG1	PG30
Score	0.00 ±0.00	3.60 ±0.57	3.40 ±0.54	3.20 ±0.44	3.00 ±0.70

p<0.05 SG vs. other groups (Kruskal-Wallis)

In relation to the villous length (Table 2), there was difference between the SHAM and the others groups (p<0.05); however, there was no difference between the CG and the post-conditioning groups (p>0.05). Relationship between score of intestinal injury and the villous length (p=0.0004 Pearson's r = -0.6771).

**TABLE 2:** Mean and standard deviation of villous length according the groups

Group	SG*	CG	PG3	PG1	PG30
Score	161.59 ±7.04	136.27 ±8.80	135.89 ±5.40	129.46 ±16.04	135.18 ±12.88

p<0.05 SG vs. other groups (ANOVA)

## DISCUSSION

After an ischemic event, reestablishment of blood inflow is the only approach to stop ischemic cascade^2^ ^,^ ^22^. However, reperfusion may end up with reactive oxygen species formation and increase in inflammatory reaction^2^ ^,^ ^22^. 

To mitigate deleterious effects of ischemia and reperfusion the use of hypertonic solutions^23^, herbals^3^ and oxygen therapy^16^ have been proposed. The currently most effective therapies are the ischemic conditioning techniques^5^ ^,^ ^7^ ^,^ ^18^ ^,^ ^21^ ^,^ ^22^. The postconditioning excels for its greatest clinical applicability compared to preconditioning, since in some clinical scenarios there is no way to predict when a tissue will be under ischemia^5^ ^,^ ^20^ ^,^ ^25^. 

The post-conditioning works primarily by reducing reperfusion injury^5^ ^,^ ^20^ ^,^ ^22^ ^,^ ^25^, Although, when analyzed after a short-term reperfusion period it shows a poor protection of structural tissue injury^1^. Therefore, this study intended to identify if different post-conditioning protocols could minimize early intestinal mucosa damage after ischemia-reperfusion syndrome.

Our data showed that all adopted post-conditioning protocols failed to minimize early intestinal mucosa damage secondary to ischemia and reperfusion syndrome. Such result does not invalidate or reduce the importance of ischemic post-conditioning^1^, yet, such fact reveals that there is no significant reperfusion structural damage after a short-term reperfusion period, possibly because there is a critical reperfusion time when it is possible to detect morphological damage.

This finding stands great importance, since during early reperfusion there is higher formation of oxygen radicals, but they are not capable to induce structural damage to the tissue^6^. On the other hand, it is possible that ischemia-reperfusion damage was mitigated by post-conditioning if cell biomarkers were analyzed, but there was not enough time of reperfusion to detect intestinal mucosa morphological damage on optical microscopy. Thus, further studies should be conducted measuring cell function markers and structure measurements to better understand postconditioning short term effects, as well if there is any difference among the adopted postconditioning protocols. 

The number of adopted postconditioning cycles did not modify the outcome. There is no consensus in literature regarding the standard pattern of post-conditioning cycles to be performed in each organ since cell metabolism might influence the number of cycles and their duration, but it is known that short cycles are more effective than long cycles^1^ ^,^ ^5^ ^,^ ^12^ ^,^ ^20^ ^,^ ^25^.

The negative result of this research does not indicate failure^1^. The acquisition of knowledge with possible errors, preventing other researchers to repeat the same method of study, justifies the publication of unfavorable results, a practice that should be estimulated^8^.

## CONCLUSION

Adopted post-conditioning protocols had no effect on protecting intestinal mucosa morphology after mesenteric ischemia and short-term reperfusion injury.
